# *In Silico* Perspectives on the Prediction of the PLP’s Epitopes involved in Multiple Sclerosis

**DOI:** 10.15171/ijb.1356

**Published:** 2017-03

**Authors:** Zahra Zamanzadeh, Mitra Ataei, Seyed Massood Nabavi, Ghasem Ahangari, Mehdi Sadeghi, Mohammad Hosein Sanati

**Affiliations:** ^1^Department of medical biotechnology. Institute of Medical Genetic, National Institute of Genetics Engineering and Biotechnology (NIGEB), Tehran, 14965/161 Iran; ^2^ Department of Neurology, Faculty of Public Health, Shahed University, Tehran, 18155/159, Iran

**Keywords:** Epitope Prediction, Human Leukocyte Antigens, Multiple Sclerosis, Molecular Mimicry, Myelin Proteolipid Protein (PLP)

## Abstract

**Background:**

Multiple sclerosis (MS) is the most common autoimmune disease of the central nervous system (CNS). The main cause of the MS is yet to be revealed, but the most probable theory is based on the molecular mimicry that concludes some infections in the activation of T cells against brain auto-antigens that initiate the disease cascade.

**Objectives:**

The Purpose of this research is the prediction of the auto-antigen potency of the myelin proteolipid protein (PLP) in multiple sclerosis.

**Materials and Methods:**

As there wasn’t any tertiary structure of PLP available in the Protein Data Bank (PDB) and in order to characterize the structural properties of the protein, we modeled this protein using prediction servers. Meta prediction method, as a new perspective *in silico*, was performed to fi nd PLPs epitopes. For this purpose, several T cell epitope prediction web servers were used to predict PLPs epitopes against Human Leukocyte Antigens (HLA). The overlap regions, as were predicted by most web servers were selected as immunogenic epitopes and were subjected to the BLASTP against microorganisms.

**Results:**

Three common regions, AA_58-74_, AA_161-177_, and AA_238-254_ were detected as immunodominant regions through meta-prediction. Investigating peptides with more than 50% similarity to that of candidate epitope AA_58-74_ in bacteria showed a similar peptide in bacteria (mainly consistent with that of clostridium and mycobacterium) and spike protein of Alphacoronavirus 1, Canine coronavirus, and Feline coronavirus. These results suggest that cross reaction of the immune system to PLP may have originated from a bacteria or viral infection, and therefore molecular mimicry might have an important role in the progression of MS.

**Conclusions:**

Through reliable and accurate prediction of the consensus epitopes, it is not necessary to synthesize all PLP fragments and examine their immunogenicity experimentally (*in vitro*). In this study, the best encephalitogenic antigens were predicted based on bioinformatics tools that may provide reliable results for researches in a shorter time and at a lower cost.

## Background

As the most common autoimmune disease of the central nervous system (CNS), multiple sclerosis (MS) is a chronic inflammatory demyelinating health problem of the CNS which leads to the neurological disability in young adults (1-3). In MS, T cells attack the myelin sheath of the neurons in the CNS, destroying the myelin and the axons (4). The Etiology of MS is unknown, but the most probable theory is based on the molecular mimicry that establishes a number of infections which can activate T cells and initiate the disease’s cascade. In this model, induced auto-aggressive T cells are followed by structural similarity between microbial epitopes and self-peptides. Therefore, Molecular mimicry can break self-tolerance and induce autoimmune responses. In T cell mediated auto-immunities, transfer of activated T cell to the animal models can lead to autoimmunity. It is noteworthy that there are some auto-antigens in the brain including Myelin Basic Protein (MBP), proteolipid protein (PLP), myelin oligodendrocyte glycoprotein (MOG), myelin associated glycoprotein (MAG), alpha-beta crystalline, and heat shock proteins that induce T cell reactivity and play a crucial role in commencing MS (5-10).

One of the brain’s auto-antigens is proteolipid protein (PLP); the most abundant autoantigen in the CNS myelin. It plays an important role in the stability of the structure and the function of the myelinated neurons (11,12). Solely, PLP composes about 50% of the total CNS myelin proteins. The protein has been isolated by Folch and Lees in 1951 (13,14). PLP is able to induce autoimmune encephalomyelitis experimentally in the rodents and non-human primates. Its post-translational modification, acylation, makes PLP an effective autoantigen (11,15).

PLP consists of 276 amino acid residues and four highly hydrophobic trans- membrane domains. In adults, PLP expression is limited to the oligodendrocyte cells (16).

PLP is a protein with a highly conserved sequence. The amino acid sequence of the PLP protein from bovine, rat, mouse, dog, and human are 99% identical, suggesting that PLP has an imperative role in the CNS (17,18). Located on chromosome 10 (Xq22.2), the 17 kbp gene of PLP protein constitutes seven exons. PLP1 encodes both PLP and its splice variant called Dm20, its 242 amino acid isoform. Dm20 mRNA lacks 105 bp of the exon 3 of the PLP mRNA; therefore, 35 amino acids of its cytosolic loop is lost (19).

Experimental autoimmune encephalomyelitis (EAE) is the animal model of MS. Based on the encephalitogenic potential in animal models and reactivity by MS T-cells, PLP epitopes were selected to induce EAE (20). Researchers have shown that PLP peptide composed of amino acids 43-64 could induce EAE in PL/J mice (21). In addition, residues 139-151, 178-191, 104-117, and 57-70 of PLP are encephalitogenic for SJL mice (22-25). Previous studies have shown that T cells can respond to the PLP40-60 (26), PLP89-106 (27), PLP30-49, PLP180-199 (28), and PLP184-209 (29). Thus, if the best PLP epitopes and significant similarity between these epitopes and some microbial epitopes could be found, molecular mimicry hypothesis could be proven.

In spite of numerous experimental studies for recognition of the epitopes related to the auto-antigenic proteins of the myelin, there isn’t a systemic method available for PLP epitope prediction as yet (29,30). Thus far, prediction methods for the recognition of the effective PLP epitopes in MS were based on the following criteria: Reports regarding encephalitogenic potential in the animal models and preferential reactivity through MS T-cells. However, several useful bioinformatics softwares have been developed to predict the most probable epitopes.

In this study, we have employed several bioinformatics tools for T-cell epitopes prediction and similarity search helped us to choose the best epitopes which can be involved in the molecular mimicry. PLP epitopes that may cause MS by molecular mimicry mechanism can be determined with these bioinformatics tools.

## 2. Objective

The purpose of the present study was to predict the auto-antigenic immunodominant epitopes of the PLP protein and characterization of the molecular/structural characteristics of which by using *in-silico* approaches as a substantial step for *in vitro* tests.

## 3. Materials and Methods

### 3.1. Sequence Retrieval, Alignment, and General Analysis

The complete annotated sequence of the human PLP and its isoforms were obtained from the NCBI database (www.ncbi.nlm.nih.gov/). Three reference sequences were obtained and were subjected to the multiple sequence alignment by using NCBI ( (http://www.ncbi.nlm.nih.gov/tools/cobalt/cobalt.cgi?link_loc=BlastHomeLink-) in order to explore the fragments that had maximum similarity. Uniprot KB (www.uniprot.org/-) and NCBI databases were used to characterize more properties of PLP. Prosite (http://prosite.expasy.org/) was applied to explore PLP motifs.

### 3.2. Analyzing Primary and Tertiary Structure of PLP

The primary protein structure of PLP (e.g. length, molecular weight (MW), isoelectric point (pI), amino acids, etc.) was arranged in Table 1 using Expasy tools (http://web.expasy.org/protparam/). Since we could not find any match in Protein Data Bank (PDB) for PLP to analyze its functional and structural motifs, we used phyre^2^ server (http://www.sbg.bio.ic.ac.uk/~phyre2/html/page.cgi?id=index) which combines learning machine methods, evolutionary information, fragment libraries and energy functions to predict protein tertiary structures and structural features. The selected model underwent energy minimization processes with the GROMOS96 method implementation of SWISS pdb Viewer software version 4 (http://www.expasy.org/spdbv/) and loops refined by Modeler software. The PLP model was validated with the help of Rampage server (31) and then visualized by the USCF Chimera software.

**Table 1:** Parameters computed using Expasy Prot Param tool.

**Accession Number (AC.)**	**PLP (**NP_001122306.1**)**
No. of amino acids	276
Mol. Wt a	29929.9
pI a	8.71
Total -R and +R a	12, 19
Inst.II a	18.92
AI, GRAVY a	91.59, 0.542

a: Abbreviations; Mol. Wt, Molecular Weight; pI, Theoretical Isoelectric Point; -R, Number of negative charged residues (Arg + Lys); +R, Number of positive charged residues (Asp + Glu); EC, Extinction Coefficient at 280 nm; II, Instability Index; AI, Aliphatic Index; GRAVY, Grand Average Hydropathicity.

### 3.3. MHC Class I and II Epitopes for T-cells

A consensus approach (based on Artificial Neural Network (ANN), Stabilized Matrix Method (SMM) and Support Vector Machine (SVM)), was used for the prediction of MHC I and MHC II binding peptides with lengths of 8-15 amino acids. T cell epitope prediction web servers were used to predict continuous epitopes. Some of these servers are:

- HLAPred (www.imtech.res.in/raghava/hlapred/ ), which is a HLA peptide binding predictor.
- IEDB (Immune-Epitope Database) server (http://tools.immuneepitope.org/-), which provides access to a reliable prediction of peptides binding to the MHC molecules**.** It estimates IC50 values for peptides binding to specific MHC molecules. Some of the most frequent alleles were used for developing T cell epitope prediction.
- CTLPred (http://www.imtech.res.in/raghava/ctlpred/), which is an ANN and SVM based CTL epitope prediction tool.
- ProPred-I (http://www.imtech.res.in/raghava/propred1/-), which predicts MHC-I binding regulation in an antigen sequence using quantitative matrices for 47 MHC-I alleles.
- ProPred (www.imtech.res.in/raghava/propred/), which predicts MHC-II binding regulation in an antigen sequence using quant itative matrices and is effective in locating promiscuous binding region used to select vaccine candidates.
- SYFPEITHI (www.syfpeithi.de), which is a predictive server featuring MHC-presented epitopes, MHC-specific anchor, and auxiliary motifs.

Furthermore, these peptides are promiscuous in their ability to bind to a variety of MHC class I and II molecules, including those most commonly found in MS patient population (HLA-A*24:02, DRB1*15, DRB1*04, DQA1*01:02/DQB1*0602, DQA1*04:01/DQB1*0402,DQA1*05:01/DQB1*0201, DQA1*05:01/DQB1*0301).

B cell epitope prediction web servers were also used to predict epitopes. A number of these servers are: IEDB (http://tools.immuneepitope.org/), BCPred (http://ailab.ist.psu.edu/bcpred/predict.html) and ABCPred (www.imtech.res.in/raghava/abcpred).

Finally, Meta prediction method was used to select the most effective epitopes. For this aim, the overlap regions which were predicted by most of the servers were selected as immunogenic regions. Web logo (http://weblogo.berkeley.edu/logo.cgi) was used to represent the conservation of the selected epitope sequences.

### 3.4. Selected Epitopes and BLASTp

After prediction of the consensus epitopes, three high ranked selected epitopes were directed to protein similarity BLAST search (BLASTp) (http://blast.ncbi.nlm.nih.gov/Blastp.cgi) for the evaluation of similar peptides reported in different bacteria, viruses, and protozoa species. The selected algorithm was blastp (protein-protein BLAST), with expecting threshold of 10, word size 3, and blosum62 matrix. Then we selected the closest hits and summarized them in an informative table.

## 4. Results

### 4.1. Alignment and General Analysis

Results obtained through NCBI database have revealed that PLP has three isoforms; isoform1 (NP_001122306.1) which is Proteolipid protein with 277 amino acid, isoform 2, DM20 (NP_955772.1) which has 35 amino acids less than PLP, and the isoform 3 (NP_001291933.1) with 222 amino acids. Their initiator methionine will be eliminated during post translation modification.

Multiple sequence alignment using NCBI showed that isoforms 2 and 3 are identical with PLP except region 117 to 152 which have been eliminated in DM20, and the region 1-55 which is lost in isoform 3.

Uniprot KB database exhibited PLP with P60201 (MYPR_HUMAN) accession number. PLP with an alternative name of Lipophilin which is the major protein from the CNS plays a vital role in the formation or maintenance of the multilamellar structure of myelin. This protein is a hydrophobic integral membrane protein containing four transmembrane regions, one cytoplasmic, and two extracellular loops. The post translational modifications of the PLP are lipidation in amino acids: 6, 7, 10, 109, 139, 141, 199 and disulfide bond formation between amino acid 184, 228 and 201, 220, respectively.

Using Prosite, it was found that PLP has two motifs at positions 28-37 (PS00575) and 228-247 (PS01004) that don’t have any defined function.

### 4.1. PLP Protein, Primary, and the Tertiary Structures

A summarized analysis of the data that was obtained by ExPASy ProtParam tool is presented in Table 1. Isoelectric point (pI) is important for estimating the solubility of the proteins. The calculated isoelectric point (pI) was 8.71. So, the PLP protein has a basic nature. The instability index (Inst.II) provides an estimation of the protein stability *in vitro*. Based on instability index, PLP could be classified as a stable protein. The high aliphatic index (91.59) shows that PLP protein is stable for a variety of temperature ranges. The Grand Average Hydropathicity (GRAVY) values showed positive results (0.542), which indicate the relatively low hydrophilicity and low interaction of the protein with the surrounding water molecules.

Physicochemical results have revealed that the most abundant amino acid residues were alanine (A) (11%), leucine (L) (11%), and glycine (G) (10%). The distribution of amino acid frequency in PLP protein showed that negative R-groups are more frequent than positive R-groups.

**Figure 1:**
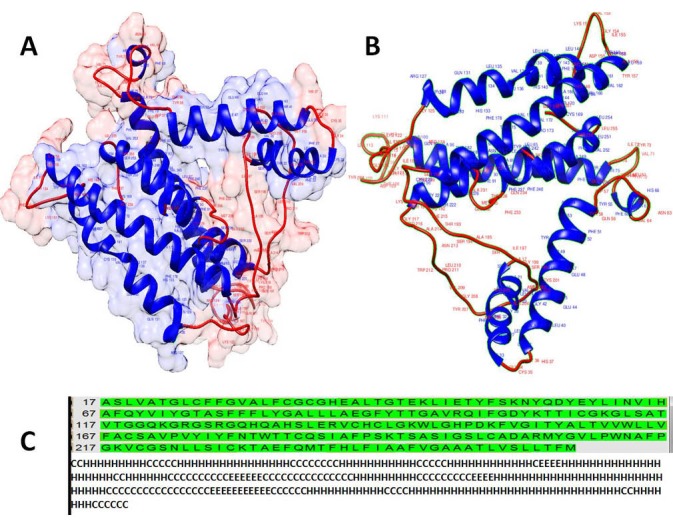
Visualizations of the predicted tertiary A: ribbon presentation with surface transparency (90%) & B: ribbon representation and secondary structure of the PLP protein (C) by Chimera software 1.8.

Furthermore, as there wasn’t any tertiary structure for this protein in the Protein Data Bank (PDB) in addition to the structural properties of this protein, we modeled this protein (Fig. 1). In order to achieve a higher quality of the modeled structure, energy minimization (E= -2432.302) and loop refinement were done. The quality of the model was validated by Ramachandran plot. Evaluation of the quality of the model by Rampage showed that the number of residues in favor, allowed, and outlier regions were 214 (89.2%of all residue)**,** 22 (9.2%), and 4 (1.7%) respectively. So, the reliability of the model by Ramachandran plot was 98.4%. Overall, these results revealed a valid modeled structure. Also, as seen in Figure 1, the model is composed of 13 coil regions (Coil), 11 helices (H), and 4 strands or beta sheet (E) substructures. A higher rate of the residue with the helix substructure reflects the transmembrane nature of the protein. As we analyzed later in the MHC prediction binding peptides, most of the epitope located in the helix regions and coil stretches may have roles in stimulation of the humoral immune response (besides cellular immune response) by antibodies.

### 4.2. MHC I & MHC II Binder Peptides (T cell Epitopes)

Applying the consensus approach results obtained for PLP interactions with the T-Cell epitopes (MHC I & MHC II binder peptides) are arranged in Table 2. This table includes peptides with higher affinities (low ranks) for the corresponding alleles while peptides with lower affinities were ignored.

**Table 2:** Predicted MHC I & MHC II binder peptides for HLA-A*24:02, HLA-DRB1*15 (subtypes), HLA-HLA-DRB1*04:01, HLA-DQA1*05:01/DQB1*03:01, HLA-DQA1*04:01/DQB1*04:02 and DQA1*01:02/DQB1*06:02. Results were arranged in their corresponding boxes based on peptides with higher scores for each allele. Arranged based on their rank. Low percentile rank = good binders.

**HLA type**	**Tools**	**Positions**
HLA-A24	BIMAS	262-270, 156-165, 72-81, 156-164
HLA-A24	HLAPred	81-89, 130-138, 37-45, 210-218
HLA-A24	IEDB	156-167, 176-187
HLA-A24	NetMHC	71-79, 231-239, 255-263, 155-163, 71-80,205-213, 155-165, 175-186, 155-166, 49-61, 48-61, 173-186, 175-188
HLA-A24	ProPred-I	262-170, 156-163
HLA-A24	SYFPEITHI	262-270, 156-165, 54-62, 59-68
HLA-A24	156-164, 262-270 CTLPred
HLA-DRB1*15	HLAPred	79-87, 70-78, 174-182, 251-259
HLA-DRB1*15	IEDB	74-88, 76-90, 249-263, 245-259
HLA-DRB1*15	ProPred	78-86, 173-181, 250-258, 69-77, 163-171
HLA-DRB1*15	SYFPEITHI	206-220, 76-90, 169-183
HLADRB1*0401	HLAPred	79-87, 70-78, 177-185, 58-66, 245-53, 251-259, 161-169
HLADRB1*0401	IEDB	251-265, 252-266, 253-267, 254-268, 255-269, 256-270, 257-271, 239-253, 241-255, 242-256, 240-255, 243-257, 249-263, 250-263, 246-260, 247-261, 248-262, 55-69, 54-68, 53-67, 173-187, 174-188, 175-189, 172-186, 171-185, 56-70, 52-66, 66-80, 65-79, 67-81, 64-78, 68-82
HLADRB1*0401	ProPred	176-184, 57-65, 244-252, 177-185, 250-258, 256-264, 171-178, 69-77, 160-168, 174-182
HLADRB1*0401	SYFPEITHI	172-186
DQA1*01:02/DQB1*0602	IEDB	241-255, 242-256, 243-257, 240-254, 244-258, 239-253, 245-259, 153-167, 75-89, 80-94,
DQA1*04:01/DQB1*0402	IEDB	77-91, 78-92, 76-90, 79-93, 80-94, 68-82, 69-83, 67-81, 75-89, 70-84, 81-95, 66-80, 239-253
DQA1*05:01/DQB1*0201	IEDB	76-90, 77-91, 75-89, 74-88, 78-92, 73-87, 79-93, 155-169, 154-168, 239-253, 238-252, 240-254, 161-175,
A1*05:01/DQB1*0301	IEDB	239-253, 240-254, 241-255, 238-252, 242-258, 237-251, 236-250, 243-257, 244-258, 235-249, 234-248, 75-89, 76-90, 77-91, 78-92, 74-88, 9-23, 10-24, 8-22

### 4.3. Selecting Epitopes for In vitro Testing

**Figure 2:**
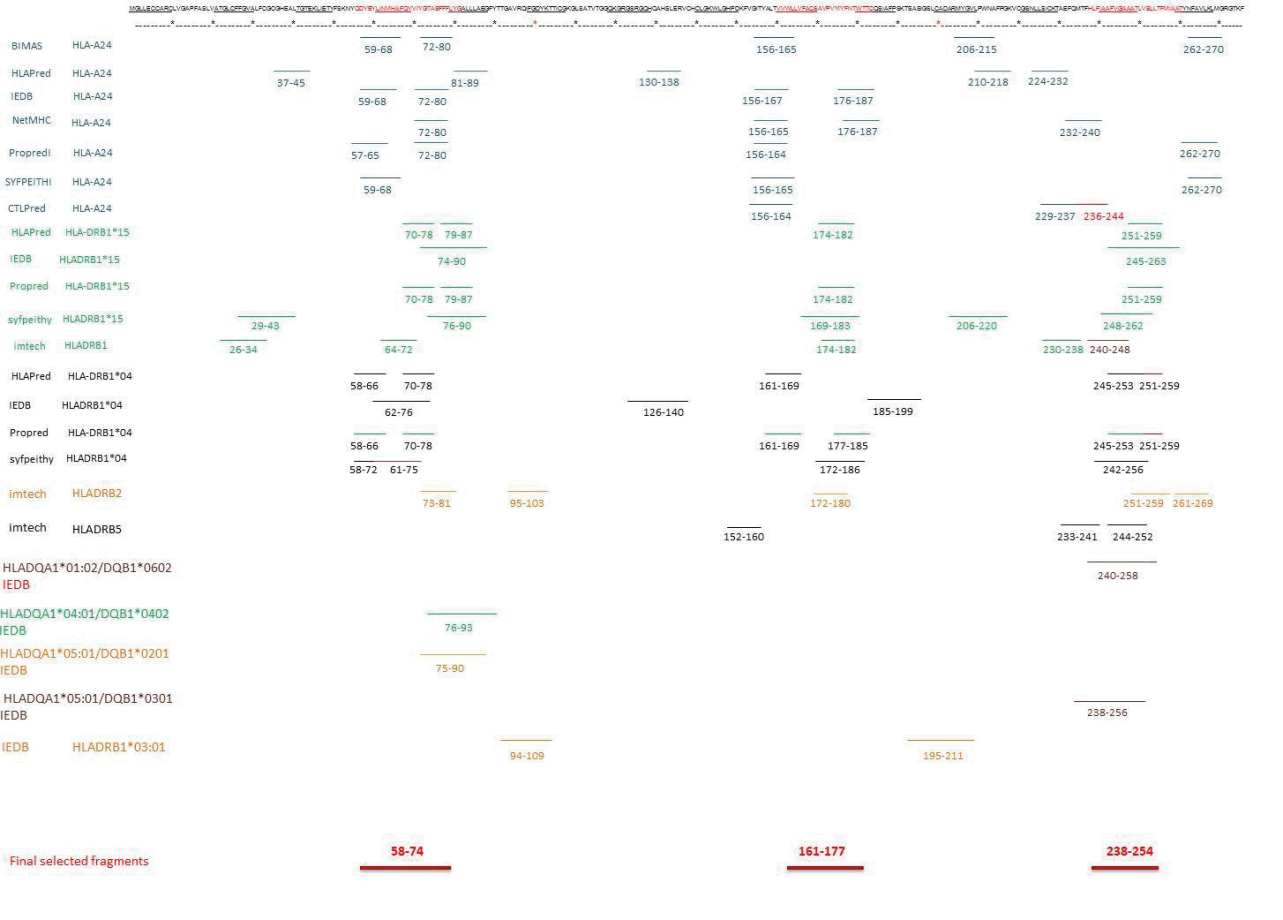
Graphical presentation of the predicted epitopes on PLP protein amino acid sequence. Three selected epitopes (AA_58-74_, AA_161-177_, and AA_238-254_) were predicted to be immunogenic regions as indicated by most database sites.

**Figure 3:**
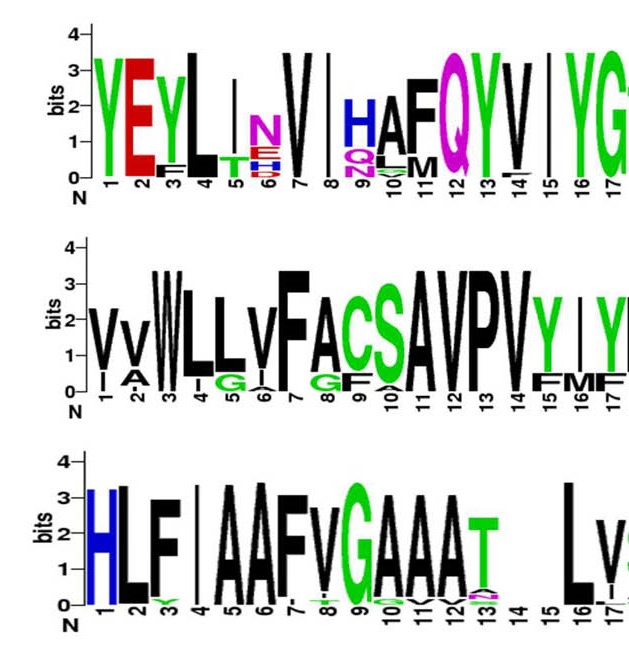
Conserved sequences inside the three selected regions. The presence of only one amino acid in each position is an indicator for the most conserved sequence and the two or more amino acids in the position show variability.

Finally, three common regions were selected as immunogenic (i.e., immunodominant) epitopes for stimulation of the both MHCI & MHCII alleles based on the predicted peptides with higher ranks for *in vitro* testing. These results were visualized as shown in Figure 2. These regions are included of the following peptides: the AA_58-74_ YEYLINVIHAFQYVIYG peptide sequence, the AA_161-177_ region: composed of VVWLLVFACSAVPVYIY amino acids, and the last region; AA_238-254_ which was composed of the peptide with subsequent amino acids HLFIAAFVGAAATLVSL. Web logo was used to evaluate the consensus sequence of the three selected zones and it was shown that these three regions are conserved sequences (Fig. 3).

The antigenic criteria proposed for the selection of these sequences were:

- The epitope region is recognized by the minimum number of fragments and the maximum properties for selection of an epitope function
- The most different algorithms in the T cell epitope prediction databases
- Identification by B cells (linear or continuous epitope prediction)
- Epitope sequences with more conserved amino acids and the most similar sequences in different organisms
- These results proposed the epitope AA_58-74_ as the highest priority and the most antigenic for MS to be tested in the experimental phase.

### 4.4. BLAST of Predicted Epitopes

Peptide sequences of the predicted epitopes were used as entry data to NCBI BLASTp search for finding the most similar peptides in bacteria, viruses, and protozoa species. BLAST results for the three candidate epitopes and protozoa with >50% similarity didn’t show any significant similarity; therefore, protozoa couldn’t induce immune cell reactivity to these epitopes of myelin PLP.

BLAST was carried out for the candidate epitope AA_58-74_ (YEYLINVIHAFQYVIYG) and bacteria with >50% similarity showed that the peptide fragment **LINVIHAFQ** is the most repeated region in bacteria and viruses.

### 4.5. Finding Similar Epitopes in Bacteria and Viruses

Peptide sequences of the predicted epitopes were searched in order to find similar peptides in bacteria and viruses using BLASTP. Results obtained for the peptides in the bacterial kingdom with more than 50% similarity to the candidate epitope, AA_58-74_ (YEYLINVIHAFQYVIYG) showed a similar peptide in the bacteria that mainly are consisted of the clostridia and mycobacteria (Table 3).

**Table 3:** BLAST results as obtained for the peptides fragments in bacterial and viral proteins similar to the analyzed epitope, AA_58-74_ in amino acid sequence.

**Protein name**	**Sequence**	**Organism**
hypothetical protein	**YEYLIN**QI**IH**S**F**	*Leptospira interrogans*
histidine kinase	**E**F**LINVI**QE**F**EKDR**QYV**	* Haemophilus infl uenza *
histidine kinase	**E**F**LINVI**QE**F**EKDR**QYV**	* Eubacterium *
histidine kinase	**E**F**LINVI**QE**F**EKDR**QYV**	* Peptostreptococcaceae bacterium *
histidine kinase	**E**F**LINVI**QE**F**EKDR**QYV**	* Haemophilus haemolyticus *
flagellar motor protein MotP	**LI**D**VIHAFQ**	* Desulfarculus sp. SPR *
predicted protein	**YEYL**DT**VIH**E**F**RHAM**QY**	* Clostridiales bacterium *
glycerol-3-phosphate ABC transporter permease	**LINVI**N**AFQ**	* Clostridium sp. LF2 *
metal dependent phosphohydrolase	**EYLI**K**VI**G**AFQ**	*Clostridium sp. CAG:230 *
hypothetical protein	**YEYLIN**N**IH**	* Salmonella enterica *
LytTr DNA-binding domain protein	**EY**MED**V**FS**IH**T**F**D**YVI**	* Enterococcus faecalis *
hypothetical protein	F**EYLI**K**VI**P**AF**	* Clostridium asparagiforme *
Wzy	**Y**Q**YLIN**G**I**R**AF**	* Cronobacter muytjensii *
RND transporter	**Y**D**YLINV**L**H**	* Acinetobacter johnsonii, A. lwoffi *
type I secretion outer membrane protein, TolC family	YD**YLINV**L**H**	* Acinetobacter johnsonii SH046 *
1 4-alpha-glucan branching enzyme GlgB	**YEY**M**INVI**	* Clostridium sp. CAG:964 *
ATP-dependent helicase/ deoxyribonuclease subunit B/ nuclease subunit B	**YE**F**LI**D-**IHAF**E	* Clostridium butyricum *
hypothetical protein	**EYL**V**NVIH**	* Spiroplasma melliferum *
hypothetical protein	**EY**-**I**D**VIHA**K**QYV**	* Paenibacillus pini *
(2Fe-2S)-binding protein	**EYL**V**N**IT**HAF**	* Pseudomonas fl uorescens *
putative N-deacylase involved in arginine and ornithine utilization	Q**YL**LD**VIH**S**F**P**Y**	* Bacillus vireti *
hypothetical protein	**NV**V**H**D**F**E**YVI**	* Vibrio sinaloensis *
hypothetical protein BN890_49820	**Y**A**YLIN**ELSGT**VI**-**AF**E**Y**	* Bacteroides xylanisolvens *
6-phosphogluconolactonase, partial	**Y**A**YLIN**ELSGT**VI**-**AF**E**Y**	* Bacteroides fragilis *
hypothetical protein	**YEYLI**Y**V**DR**IHA**	Bacteroides
hypothetical protein	**LI**D**VIHA**IHFI**I**	* Acinetobacter beijerinckii *
tRNA pseudouridine synthase A	**YEYLIN**-TR**AF**NPA**QY**	* Mycoplasma pneumonia *
hypothetical protein	**YEYLINV**	* Lachnospiraceae bacterium *
binding--dependent transport system inner membrane component family protein	**I**S**VIHAFQ**AFDMV**YV**	* Mycobacterium avium subsp. avium 2285 *
sugar ABC transporter permease	**I**S**VIHAFQ**AFDMV**YV**	* Mycobacterium colombiense CECT 3035 *
MULTISPECIES: ABC transporter permease	**I**S**VIHAFQ**AFDMV**YV**	* Mycobacterium avium complex (MAC) *
hypothetical protein	**YEYLI**Y**V**DR**IHA**	* Bacteroides sp. CAG:661 *
cytochrome oxidase assembly protein	**EY**F**INVIH**L**F**	Leptospira
ABC transporter permease	**I**S**VIHAFQ**AFDMV**YV**	* Mycobacterium tuberculosis/ colombiense/ intracellulare/ avium complex *
6-phosphogluconolactonase	**Y**A**YLIN**ELSGT**VI**-**AF**E**Y**	* Bacteroides sp. D2 *
aromatic amino acid aminotransferase	**EYLI**KMMKK**IH**—**QYVI**	* Bacterium LF-3 *
ac70-like protein	**Y**N**YL**K**NVIHA**	* Clanis bilineata nucleopolyhedro virus *
unnamed protein product	**EYLINVI**	* Pseudomonas phage OBP *
hypothetical protein SP126_00040	**EY**VR**NV**A**HAF**DN**VI**	* Salmonella phage FSL SP-126 *
putative membrane protein	**LI**K**VIH**GLE**Y**I**I**	* Enterobacteria phage *
hypothetical protein SPC35_0038	**LI**K**VIH**GLE**Y**I**I**	* Salmonella phage Spc35 *
Flavodoxin	**YL**----**HAF**E**YVI**	* Bacillus phage *
multifunctional replicase, partial	F**EYL**L**NV**F**HA**	*Chara australis virus *
ORF140	**YEY**EVKV**V**QL**FQY**I	* Cydia pomonella granulovirus *
m152 protein	**YEYL**M**NV**FK**A**GRPII**F**E**Y**	* Murid herpesvirus 1 *
hypothetical protein BpV1_188c	**YEYL**L**N**-**I**KTY**QY**	* Bathycoccus *
Endonuclease	**YEYLI**SN**IH**	* Enterobacteria phage HK629 *
Protein MGF 505-7R	**E**F**LIN**I**IH ,** D**YLI**	African swine fever virus * Malawi LIL 20/1 *
thymidylate kinase	**YEYLIN**	* Halovirus HVTV-1 *
regulatory protein	**I**D**VIHA**IE**Y**I	* Escherichia phage 1720a-02 *
RNA polymerase subunit RPO147	**YEYLIN**	* Deerpox virus *
spike glycoprotein	**YEY**FN**N**-**IHAF** **EYLI**	* Canine coronavirus *
spike protein, partial	**YEY**FN**N**-**IHAF**	* Alphacoronavirus 1 *
envelope glycoprotein	**LI**-**VIHA**L**QY**	* Human immunodefi ciency virus 1 *
putative ankyrin repeat protein	**LINV**EQN**I**-**F**E**YVI** F**EYLI**	* Acanthamoeba polyphaga mimivirus *
putative NAD+ dependent DNA ligase	**YEY**I**IN**ILN**A**	* Amsacta moorei entomopoxvirus *
DNA primase	**E**C**LIN**KRSRD**VIHA**I**FQ**	*uncultured Mediterranean phage uvMED *
Spike glycoprotein	**YEY**FN**N**-**IHAF**	*Feline coronavirus *
spike protein/ S protein	**YEY**FN**N**-**IHAF**	* Canine coronavirus *
CI repressor	**I**D**VIHA**IE**Y**	Shigella phage
regulatory protein	**I**D**VIHA**IE**Y**	Salmonella phage

Investigation on the similarity between the epitope AA_58-74_ and viruses has led us to a region in the spike protein of the alphacoronavirus 1, Canine coronavirus, and Feline coronavirus (Table 3). Spike protein/S protein or spike glycoprotein is a major protein in the pathogens of the severe acute respiratory syndrome (SARS) associated with coronavirus (32).

These results suggest that the cross reaction of the immune system with the PLP may have originated from a bacterial or viral infection and therefore, genetic makeup (the presence of the susceptible alleles in one person) and exposure to foreign organism contribute to the increasing risk of auto-immune diseases like MS.

## 5. Discussion

The causes of MS are unknown, but the most probable theory is suggested to be through a molecular mimicry process via infections that activation T cells. T cell reactivity to brain auto-antigens, particularly myelin antigens, plays a crucial role for commencing of the MS (5,6).

Molecular mimicry is also known as “cross-reactivity” between self-antigens and immunodominant epitopes of the pathogens. Cross-reactivity can be the cause of many autoimmune disorders such as myasthenia gravis which occurs due to detectable antibodies produced against the human acetylcholine receptor. Cross-reactivity of the self-epitope with the antibodies produced against herpes simplex virus glycoprotein D suggests that the virus is associated with the initiation of myasthenia gravis (33).

Schloot *et al.* in 2001 have shown that isolated T cells from type 1 diabetes patients which are reactive to glutamic acid decarboxylase65 (GAD65) cross-react with the Coxsackie virus protein P2C and pro-insulin protein (34).

There is a significant association between parvovirus B19 cross-reactivity and several auto-immune disorders including rheumatoid arthritis, systemic lupus, anti-phospholipid syndrome and systemic sclerosis (35). Poole *et al*. in 2006 have demonstrated that antibodies produced against Epstein-Barr virus nuclear antigen-1 (EBNA-1) cross-react with lupus-associated auto-antigens (36).

Therefore, molecular mimicry between the host and microbial proteins seems to be the main mechanism involved in the induction of autoimmunity and MS. There are numbers of evidence which prove the role of molecular mimicry in the mechanism of MS incidence. Due to the abundance of PLP in CNS myelin and its restriction to the CNS, it has been the most important candidate in the pathogenesis of MS.

The only known structure in the PDB was a monomeric peptide (KLIETYFSK) in complex with HLA-A0301 (PDB ID 2XPG), therefore, we modeled the protein.

We have taken a systematic approach based on available bioinformatic tools and three fragments were selected as the immunogenic epitopes. Whitham *et al.* (1991) have already shown that residue 43-64 of PLP could induce EAE in PL/J mice. Other researchers have shown PLP peptides 139-151, 178-191, 104-117, and 57-70 are encephalitogenic for SJL/J mice (22-25). In 1993 Pelfrey *et al.* have suggested that T cells can respond to the PLP 40-60 fragment. Greer *et al.* (20004) synthesized the fragments 41-58, 95-114, 178-191, 184-199, 190-209, 100-119, and 139-151 and treated MS patients’ PBMC with such synthetic peptides. Although the result was the proliferation in response to the peptide fragments 184-199 and 290-209 (29), they synthesized the fragments randomly without considering continuous epitopes processing ways in proteasome system of antigen presenting. Their prediction methods for recognition of the effective PLP epitopes in MS were based on the following criteria: Reports of encephalitogenic potential in animal models and preferential reactivity by MS T-cells.

The immune system has an unavoidable role in the pathogenesis of MS through the major histocompatibility complex (MHC) class II (15). Moreover, Continuous fragments which can be recognized by T cells and were processed by proteasome complex of antigen presenting cells are presented by MHCII. Besides, it was proposed that MHC class I has an important role in the initiation of MS too, because (i) CD8+ T cells are the most common cells in the lesions, (ii) MHC I molecule is up-regulated in the induced EAE mice using TMEV (37,38).

Some MHC I and II alleles have been associated with MS and each class contributes to the risk of the disease, such as HLA-A24, HLA-DRB1*15, HLA-DRB1*04, HLADRB1*03:01, HLADRB2, HLADQA1*01:02/DQB1*0602, HLADQA1*04:01/DQB1*0402, HLADQA1*05:01/DQB1*0201, and HLADQA1*05:01/DQB1*0301. While it was shown that HLA-A2 has a protective effect against MS (29,39,40). Selection of the different HLAs involved in the MS initiation has improved the prediction validity in order to use results at the population scale.

As mentioned above, previous studies have experimentally investigated the immunogenic epitopes of the PLP by (i) induction of EAE with overlapping peptides or (ii) their effects on patients’ T cells proliferation (29,30). Also, some reports have focused on homology between brain auto-antigens and microorganisms which may have a role in MS initiation through molecular mimicry mechanism (9). Nowadays it is possible to predict immunogenic epitopes *in-silico* without a need for the synthesis of each fragment and test them. Such systematic approaches are mainly needed for the prediction of the immunogenic epitopes which have high homology with the microorganisms.

As indicated above, in the initiation phase of this research, the so-called “*in-silico* Phase”, was done through comprehensive predictions by bioinformatics tools despite other published articles. PLPs antigenic epitopes sequences were predicted as the myelin-specific protein with common antigenicity among the PLPs and a wide range of membrane protein as pathogen epitopes.

On the other hand, several useful bioinformatics software has been developed to predict the most probable epitopes. In this study, the best encephalitogenic epitopes were predicted according to the bioinformatics tools that may provide reliable results for researches. In this research, fragments with the best scores were selected, so they were aligned together in order to choose the most overlapping regions.

BLAST results for different predicted peptide epitopes have revealed high similarity (>50% in most cases) between human PLP to a number of bacterial and viral components. Most of the BLAST hit results were related to the membrane proteins (such as; Transporters), permeases, transferases (Histidine kinase), spike protein etc. This shows the epitopes of PLP may induce MS through a molecular mimicry mechanism.

As it is seen in Table 3 (BLASTP results), the candidate epitope showed a high similarity with several antigenic bacterial and viral membrane proteins such as spike protein. These findings can provide evidence for the role of molecular mimicry in the initiation of MS. Based on this theory and the results of this research, the cross-reaction between PLPs and bacterial or viral epitopes can ignite a cascade of immune responses which could lead to MS.

Based on the results of this work PLP_58-74_ could be a candidate as an immunodominant epitope of the PLP. We tested our hypothesis experimentally, therefore, patients and healthy individuals’ peripheral blood mononuclear cells (PBMCs) were treated by PLP_58-74_ and its proliferative effects were evaluated through proliferating cell nuclear antigen (PCNA) gene expression changes assessment by real time PCR and immunocytochemistry assay. Experimental results showed that PLP_58-74_ could induce proliferation in patient’s PBMCs while it did not influence the PBMCs of the healthy individuals (41)_._

Massilamany *et al*. (2010) showed that there is a cross reaction between PLP_139-151_ and *Acanthamoeba castellanii* (ACA)_83-95_ and both of them induce EAE in SJL/J mice. They observed that both epitopes (PLP_139-151_ and ACA_83-95_ ) could stimulate proliferation of the T cell derived from lymph nodes (7). Wegmann *et al.* (2014) observed that EAE mice induced using PLP_139-151_ could be treated by modified ACA_83-95._ They stated that ACA_83-95_ prevents the development of rEAE (42). Also, in 2013 Badawi and Siahan showed that multivalent bifunctional peptide inhibitors (MVB_MOG/PLP_) can suppress MOG38-50- and PLP139-151-induced EAE (43).

Olson *et al.* (2001) engineered a nonpathogenic TMEV variant to encode the PLP_139-151_ epitope for the assessment of molecular mimicry potential in the initiation of MS. They showed that there is a cross reaction between pathogenic TMEV, PLP139-151 epitope, and *Haemophilus influenza* which could induce EAE in SJL/J mice (44).

Many microorganisms have been suspected of involved in MS. Greene, (2008) has indicated that *Mycobacterium avium and Haemophilus influenza* can induce EAE in dTgH2Aneg mice (45). Also, Westall (2006) has stated that gut bacteria such as mycobacterium, clostridium, salmonella etc., might be associated with MS initiation (46).

Among the main achievements of the present study was the modeling of PLP. As there wasn’t any tertiary structure of this protein available in the Protein Data Bank (PDB), for characterizing the structural properties of this protein we modeled this protein.

The results of this study contribute to a better understanding and clarification of the mechanism of the disease and causes of MS. These results can be used to prevent the initiation of MS, immunotherapy, and individual medicine in future.

## 6. Conclusion

Herein, we tried to find immunodominant epitopes for cytotoxic and helper T cells based on susceptible alleles to MS, checking their matches in microbial components and to clarify some of the structural characteristics of the PLP as an important protein in MS pathogenesis. By reliable and accurate prediction of the consensus epitopes, it is not necessary to synthesize all PLP fragments and examine their immunogenicity experimentally (*in vitro*). Therefore, researchers can achieve better results in a shorter time and at a lower cost. Besides, they could check if the consensus predicted epitopes have a cross reaction with the foreign particles, immunotherapeutic regimens, and gain a better understanding of the mechanism of MS diseases. This approach allows the development of peptide-based pharmacotherapy for MS and promises for personalized medicine in future.

## Acknowledgments

This work was supported by a grant from the National Institute of Genetic Engineering and Biotechnology (NIGEB) of Iran.
